# Photophysics of Fluorescent Contact Sensors Based on the Dicyanodihydrofuran Motif

**DOI:** 10.1002/cphc.202000860

**Published:** 2020-12-04

**Authors:** Tomislav Suhina, Daniel Bonn, Bart Weber, Albert M. Brouwer

**Affiliations:** ^1^ van ‘t Hoff Institute for Molecular Sciences University of Amsterdam Science Park 904 1098 XH Amsterdam The Netherlands; ^2^ Institute of Physics University of Amsterdam Science Park 904 1098 XH Amsterdam The Netherlands

**Keywords:** contact mechanics, fluorescence, probe, transient absorption, viscosity

## Abstract

Fluorescent molecular rotors have been used for measurements of local mobility on molecular length scales, for example to determine viscosity, and for the visualization of contact between two surfaces. In the present work, we deepen our insight into the excited‐state deactivation kinetics and mechanics of dicyanodihydrofuran‐based molecular rotors. We extend the scope of the use of this class of rotors for contact sensing with a red‐shifted member of the family. This allows for contact detection with a range of excitation wavelengths up to ∼600 nm. Steady‐state fluorescence shows that the fluorescence quantum yield of these rotors depends not only on the rigidity of their environment, but – under certain conditions – also on its polarity. While excited state decay via rotation about the exocyclic double bond is rapid in nonpolar solvents and twisting of a single bond allows for fast decay in polar solvents, the barriers for both processes are significant in solvents of intermediate polarity. This effect may also occur in other molecular rotors, and it should be considered when applying such molecules as local mobility probes.

## Graphical abstract

**Either way**: Molecular fluorescent rotors based on the DCDHF motif can decay via twisting of two different bonds, with different sensitivities to solvent polarity. An implication of this mechanistic complexity is that their fluorescence intensity may depend on viscosity and polarity. Nonetheless, two probes that differ only in their conjugation length can both be used fruitfully to monitor viscosity and mechanical contact. The high local pressure in the mechanical contact leads to confinement

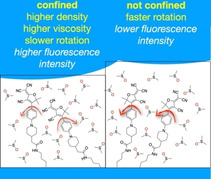

## 1 Introduction

**Scheme 1:**
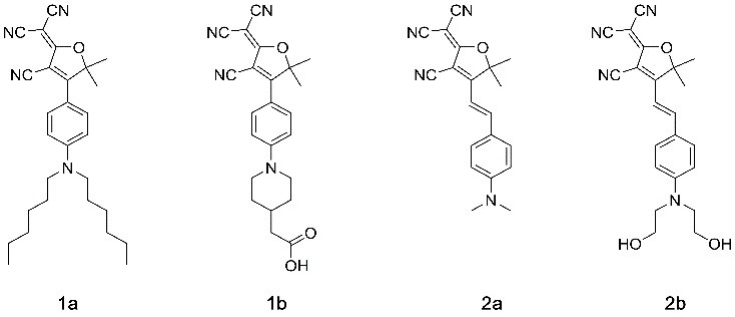
Molecular structures of compounds used in this study. For spectroscopic studies model chromophores **1 a** and **2 a** were used; **1 b** and **2 b** were used to prepare the surface‐bound versions of the same chromophores.

Molecular rotors are fluorescent molecules in which a large amplitude motion allows for the fluorescent excited state to decay via a low energy barrier to a twisted intermediate or directly to the ground state through a conical intersection.[^1^, ^2^, ^3^, ^4^, ^5^, ^6^, ^7^, ^8^] When this motion is suppressed due to limited mobility of the environment of the molecule, fluorescence can be restored. This is the working principle for a large number of viscosity sensors,[^9^, ^10^, ^11^, ^12^, ^13^] and, for example, fluorescent probes for DNA, which are based on intercalation as a means of restricting motion.^[14]^ In our own work we have applied this concept to the detection of mechanical contact using fluorescence microscopy.[^15^, ^16^, ^17^, ^18^] Our rotor of choice was the dicyanodihydrofuran chromophore **1** (DCDHF; Scheme 1), which had been reported by Twieg and Moerner, initially for non‐linear optical applications,^[19]^ and later as a single‐molecule dye.^[20]^ Our reasons for selecting this dye were the known photostability, and the favorable spectroscopic properties for fluorescence microscopy using the standard 488 nm excitation wavelength.

**Scheme 2:**
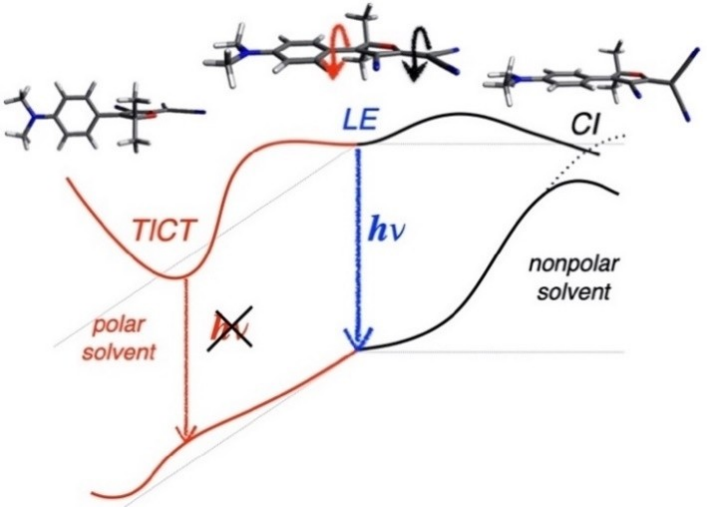
Excited‐state deactivation pathways of **1**, illustrated with calculated model structures. In non‐polar solvents twisting of the C=C bond is preferred (black arrow), leading to a conical intersection; in polar solvents a twisted intramolecular charge transfer state (TICT) is formed (red arrow).

The mechanism of the nonradiative decay of **1** was found to be unusual (Scheme 2): in nonpolar solvents rotation occurs about the exocyclic C=C double bond, leading to a conical intersection and direct decay to the ground state. In polar solvents, a twist about the single bond between the rings leads to a dark state, that we could identify using time‐resolved infrared spectroscopy, and indirectly via delayed fluorescence.^[21]^ In solvents of intermediate polarity, a transition occurs between the two modes of decay, and the fluorescence is relatively intense. This is an important point to consider for applications of this type of viscosity sensor,^[22]^ and it is further explored in this paper. In the present work we also directly demonstrate the presence of the non‐fluorescent intermediate state in several solvents using visible time‐resolved absorption spectroscopy. Finally, we extend the range of DCDHF rotors for contact sensing with a red‐shifted family member **2** that can be excited in the range 500–600 nm. The photophysical behavior of **2** is similar to that of **1** and it also performs well as fluorescent probe for viscosity and contact.

## 2 Results and Discussion

### 2.1 Transient Absorption Spectroscopy

**Figure 1:**
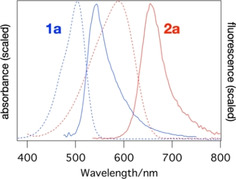
Scaled absorption (dashed) and emission spectra (full lines) of **1 a** and **2 a** in DMSO.

The excited‐state deactivation of **1** was studied using transient absorption measurements in solvents of low (*n*‐hexane and toluene), medium (ethyl acetate=EtOAc) and high polarity (CH_3_CN and DMSO). For **2 a** we present data in MeOH, EtOAc and DMSO. Representative absorption and fluorescence spectra are shown in Figure 1. Additional spectra of **2** in toluene, EtOAc and DMSO are shown below in Figure 5.

**Figure 2:**
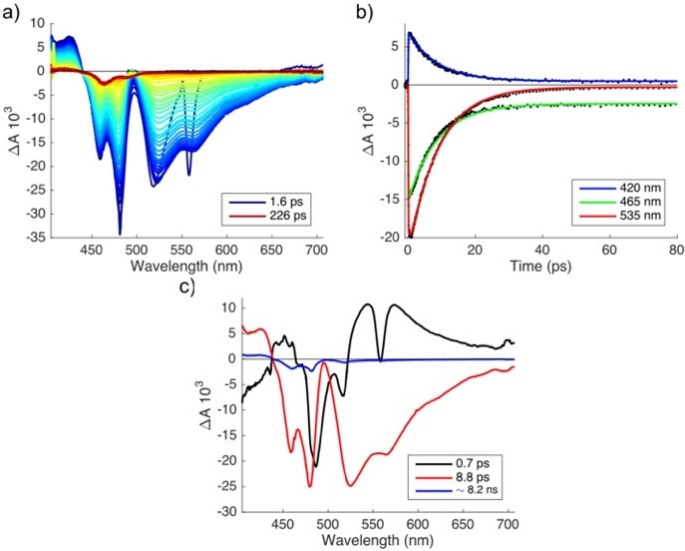
Vis‐pump/vis‐probe measurements for **1 a** in n‐hexane: a) transient spectra at different delay times; b) selected time traces (black markers) and fits (colored lines) produced by compartmental global analysis; c) decay‐associated difference spectra.

In transient absorption data for **1 a** in *n*‐hexane (Figure 2) we can distinguish three spectral regions: ground‐state bleach (GSB) corresponding to the ground state absorption band (∼450–500 nm), stimulated emission (SE), in the same spectral range as spontaneous fluorescence (∼500–700 nm), and excited‐state absorption (ESA) at ∼405–440 nm. ESA, SE and most of GSB decay with a time constant of ∼9 ps. At the end of the measurement time window of 3.6 ns, a long‐lived component remains, with a spectrum resembling GSB. Global analysis of the transient data matrix^[23]^ produces three time constants, τ_1_=0.7 ps, τ_2_=8.8 ps and τ_3_∼8.2 ns. The latter is not reliable because of the limited time range probed in the present experiment. The actual lifetime may well be much longer. The reconstructed decay‐associated difference spectra (DADS) are shown in Figure 2c. We associate τ_1_<1 ps with the vibrationally hot locally excited (LE*) state, τ_2_=8.8 ps with the relaxed locally excited (LE) state and τ_3_ with a long‐lived transient, formed with a yield of 6–10 %. The nature of the long‐lived species is at present unknown. From the observation that the absorption spectrum of the sample had not changed much after the pump‐probe experiments we infer that it is indeed a transient intermediate, and not a permanent photoproduct.

Transient absorption spectra of **1 a** in toluene are similar to those in *n*‐hexane (Figure S2, see the Supporting Information). They show ESA (415–450 nm), GSB (450–505 nm) and SE (510–680 nm). Ground state bleach and stimulated emission signals are red‐shifted relative to those of **1 a** in hexane, because toluene is more polar.[^24^, ^25^] From global analysis of the transient data matrix we obtain two time constants τ_1_=2.7 ps and τ_2_=136 ps. The DADS for the sequential evolution [Eq. (1)] are shown in Figure S2c.

(1) $$\mathrm{LE}{}^{*}\to \mathrm{LE}\to S{}_{0}$$

The time constant τ_1_=2.7 ps of the LE* state is similar to the solvent reorientation time reported for toluene.^[26]^ The second component corresponds to the decay of the relaxed LE state via twisting of the C=C bond, leading to the ground state without any intermediate. The time constant is in good agreement with the results of IR transient absorption and fluorescence measurements.

The decays in toluene, as shown in Figure S2b, are much slower than those in hexane. We attribute this mainly to the increase of the rotation barrier in the solvent of higher polarity (Scheme 2).

**Figure 3:**
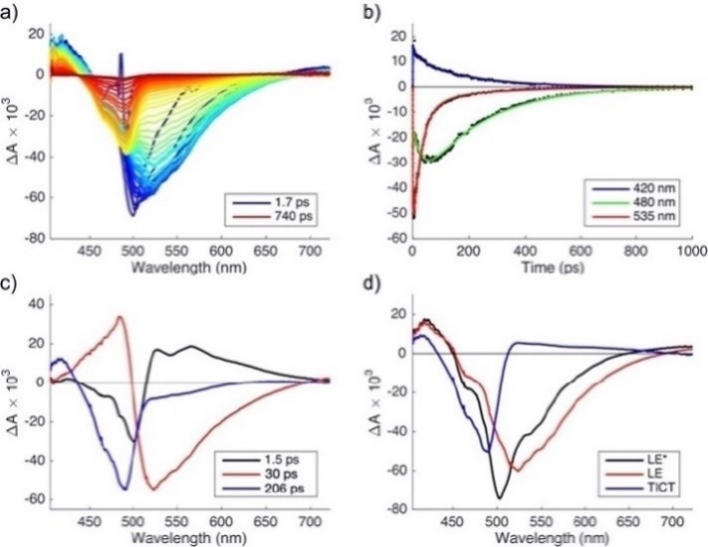
Vis‐pump/vis‐probe probe measurements for **1 a** in EtOAc: a) selected transient spectra; b) selected time traces (black markers) and fits (colored lines) from compartmental global analysis; c) decay‐associated difference spectra (with time constants); d) species‐associated difference spectra.

The excited‐state dynamics of **1 a** in EtOAc are different from those in toluene and hexane. As we have inferred in ref. [21] from the bi‐exponential fluorescence decay, an intermediate non‐fluorescent species is formed, which can convert back to the fluorescent LE state, leading to delayed fluorescence. Here we directly detect this intermediate, that we associate with the TICT species, in the transient absorption spectra (Figure 3).

A time trace measured at 480 nm (Figure 3b) shows a pronounced increase of −ΔA at early times that correlates with the decay of the signal measured at 535 nm (stimulated emission), and indicates formation of the intermediate species. Global analysis of the transient data matrix produces time constants τ_1_=1.5 ps, τ_2_=30 ps and τ_3_=206 ps. The reconstructed DADS are shown in Figure 3c. As in the other solvents, τ_1_ is associated with solvation and vibrational relaxation. The second component (τ_2_) shows a large negative amplitude in the SE region, and a large positive amplitude is associated with this component in the GSB region (Figure 3). The DADS associated with the longest time constant (τ_3_) shows a large amplitude in GSB, and a smaller amplitude in the SE region. The values of τ_2_ and τ_3_ are in good agreement with those obtained from fluorescence decay measurements at room temperature in ref. [21].

**Scheme 3:**
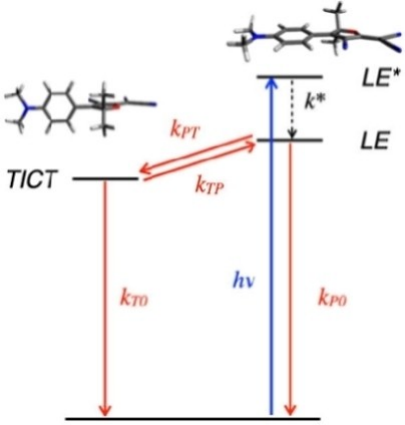
Kinetics of **1 a** as observed e. g. in EtOAc.

We model the transient data matrix according to the processes depicted in Scheme 3. In order to obtain species‐associated difference spectra we fixed the values of the rate constants (except k*, which was optimized) to values previously obtained from fluorescence decay measurements^[21]^ at room temperature: k*=0.69×10^12^ s^−1^ (optimized parameter), k_PT_=2.53×10^10^ s^−1^ (fixed parameter), k_TP_=4.78×10^9^ s^−1^ (fixed) and k_T0_=5.84×10^9^ s^−1^ (fixed). The resulting species‐associated difference spectra are shown in Figure 3d and selected time traces with fits are shown in Figure 3b. The spectrum of the TICT state appears to be a broad ESA band, largely overlapping with the GSB, and not showing any SE.

**Figure 4:**
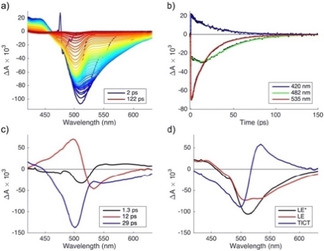
Vis‐pump/vis‐probe probe measurements for **1 a** in DMSO: a) selected transient spectra; b) selected time traces (black markers) and fits (colored lines) produced by compartmental global analysis; c) decay‐associated difference spectra (with time constants); d) species‐associated difference spectra.

For **1 a** in DMSO, formation of the TICT state was demonstrated in ref. [21] using time‐resolved infrared (TRIR) spectroscopy. In the visible TA spectra (Figure 4) we see the signatures of the LE* and LE states, as in the other solvents, and also the rise of the TICT state. Global analysis produces time constants of τ_1_=1.3 ps, τ_2_=12 ps and τ_3_=29 ps, and these values are in excellent agreement with those obtained in our previous work.^[21]^ The reconstructed DADS and the SADS for the sequential model, extended with the TICT state [Eq. (2)] are shown in Figures 4c and 4d, respectively. The rate constants are: k*=0.77×10^12^ s^−1^, k_PT_=3.4×10^10^ s^−1^ and k_T0_=8.3×10^10^ s^−1^, respectively.

(2) $$\mathrm{LE}{}^{*}\to \mathrm{LE}\to \mathrm{TICT}\to S{}_{0}$$

The spectral features are similar as observed above, confirming that the intermediate observed in the EtOAc by means of delayed fluorescence and TA, and in DMSO by TRIR and vis TA, is the same. The main difference in photophysical dynamics in EtOAc and DMSO is that the TICT state does not revert to the LE state. This is expected because the TICT state has a larger dipole moment (μ_TICT_>μ_LE_) and is more stabilized in the more polar solvents.

Excited‐state dynamics of **1 a** in acetonitrile (Figure S3) are similar to those in DSMO, but faster. Global analysis produces time‐constants τ_1_=0.5 ps, τ_2_=6.2 ps and τ_3_=10.4 ps. The reconstructed DADS (Figure S3c) are similar to those obtained in DMSO and indicate sequential dynamics of the TICT state formation (eq. 2). The rate constants are: k*=2.2×10^12^ s^−1^, k_PT_=1.6×10^11^ s^−1^ and k_T0_=9.6×10^10^ s^−1^, respectively. All three time constants are smaller than those in DMSO due to the lower viscosity of acetonitrile (*η*=0.36 mPa s *vs. η=*2.0 mPa s for DMSO).

In summary, in this section we have identified the spectral signatures of the transient species of **1 a**. The locally excited state spectrum reveals ESA, GSB and SE, and the TICT intermediate is characterized by GSB and a weak, broad ESA. The results fully support the previously proposed mechanism of the excited state decay via two different pathways.

### 2.2 Photophysical Properties of Extended DCDHF 2

**Figure 5:**
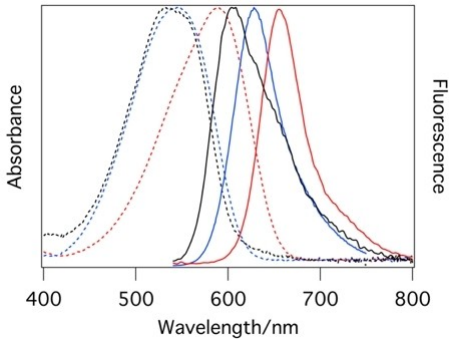
Absorption (dashed lines) and fluorescence spectra (solid lines) of **2 a** in toluene (black), EtOAc (blue) and DMSO (red).

In order to extend the working range of the DCDHF based contact sensors towards longer wavelengths, which is attractive for microscopy applications because background luminescence is typically smaller in the red, we investigated molecular rotor **2 a**, which was synthesized according to procedures adapted from the literature.[^27^, ^28^] This is one of the few molecules in the DCDHF family that was reported to show only weak fluorescence in solvents of low viscosity.[^25^, ^29^, ^30^] Apparently, the tendency for the excited states to decay via rotational motion pathways decreases when the aromatic system is extended.^[31]^ Absorption and fluorescence spectra of **2 a** in some representative solvents are shown in Figure 5. Fluorescence lifetimes and quantum yields are reported in Table 1. Fluorescence decay times in other solvents are reported in Table S1 (in the Supporting Information). It is noteworthy that, as in the case of **1 a**,^[15]^ the decays are non‐exponential in solvents of intermediate polarity.

**Table 1:** Fluorescence and transient absorption data of **2 a** in representative solvents.

	Transient absorption^[a]^			TCSPC^[a]^	
Solvent	τ_1_ [ps]	τ_2_ [ps]	τ_3_ [ps]	τ_f_ [ps]	Φ_f_^[b]^×100
EtOAc	1.2	22	236	n.a.	4.5
MeOH	0.6	5.2	29	28	0.6
DMSO	0.7	2.9	86	70	1.4

[a] τ are decay time constants. [b] Fluorescence quantum yield.

**Figure 6:**
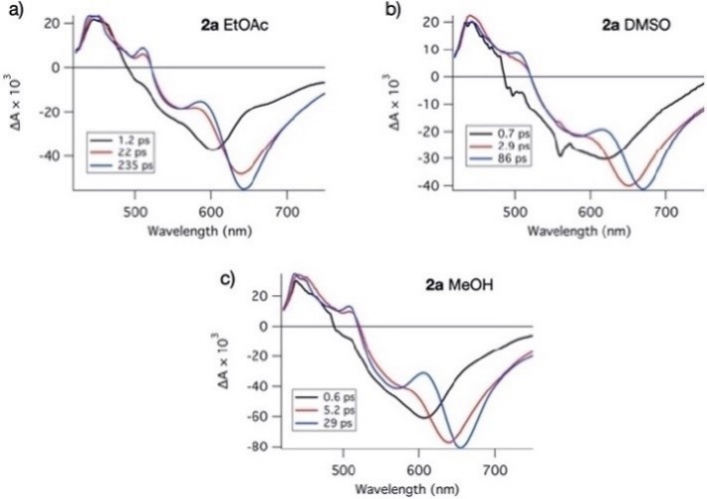
Evolution associated difference spectra (EAS) of **2 a** in EtOAc (a), DMSO (b), and MeOH (c).

We measured vis‐pump vis‐probe transients of compound **2 a** in EtOAc, MeOH and DMSO. Evolution‐associated difference spectra (sequential model) are shown in Figure 6a, b and c. Three time constants are needed to describe the spectotemporal evolution. In all cases τ_1_∼1 ps corresponds to vibrational relaxation. The intermediate τ_2_ of the order of a few ps in the two polar solvents can be due to solvation, leading to a red shift of the SE band. In EtOAc, on the other hand, τ_2_∼22 ps is too long to be explained in this way. Since EAS2 and EAS3 in this case are very similar, it is conceivable that reversible interconversion between the fluorescent LE state and the non‐fluorescent TICT state occurs, as in **1 a**.^[21]^ The third (and slowest) component is attributed to the excited‐state population decay. The decay time of this component is in good agreement with the time constant obtained by measuring fluorescence decays using time correlated single photon counting. Since the SE band is observed prominently in all EAS, it appears that the non‐fluorescent TICT state is not observed in **2 a**.

### 2.3 Solvent Polarity Effect on the Non‐Radiative Decay of 1 and 2

For **1 a** we previously demonstrated that two different decay pathways are available, involving rotation of the exocyclic C=C bond and rotation of the C−C bond between the two rings.^[21]^ The barrier of the former process is low in nonpolar solvents and increases with solvent polarity, the latter has a lower barrier in more polar solvents. A result of this is that in an intermediate solvent polarity range, neither barrier is very low, and the excited state lifetime is relatively long and the fluorescence quantum yield relatively high. In this section we investigate the fluorescence intensity of **1 a** and **2 a** in solvent mixtures giving a smooth transition between polar and nonpolar media. We choose toluene as the non‐polar co‐solvent, and examined the fluorescence response by titrating toluene solutions of **1 a** with MeOH, DMSO and MeCN as polar co‐solvents. For **2 a** a similar experiment was carried out using toluene and MeCN. Surely, solvent mixtures are complex, and the influence of specific interactions/solvation will be different depending on the system studied.[^32^, ^33^, ^34^, ^35^] We will simply assume that the dielectric constants of the solvent mixtures are the weighted averages of those of the components.

**Figure 7:**
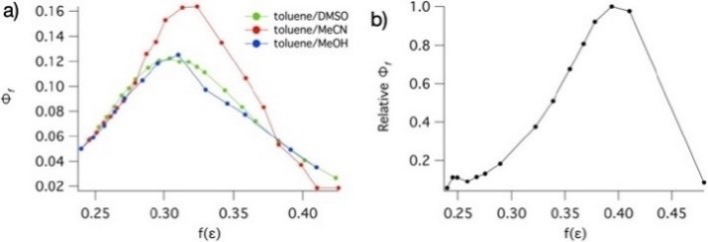
a) Fluorescence quantum yields of **1 a** (relative to the value in toluene) in solvent mixtures with different polarities with polarity functions *f*(ϵ)=(ϵ‐1)/(2ϵ+1). b) Relative fluorescence quantum yields of **2 a** in mixtures of toluene and acetonitrile.

Fluorescence quantum yields of **1 a** plotted as a function of the Onsager dielectric function of the solvent *f*(ϵ) are shown in Figure 7a. Starting from toluene, fluorescence quantum yields show an initial increase with an addition of the polar co‐solvent because the barrier for twisting the C=C bond increases. As the environment polarity increases beyond *f*(ϵ)∼0.32, the deactivation pathway through formation of the TICT state becomes favorable. This causes fluorescence quantum yields to reach a maximum and decrease as the TICT deactivation pathway becomes more accessible with the increase in solvent polarity. For comparison, in neat EtOAc (*f*(ϵ)=0.38) Φ_f_=0.029.^[21]^ We observe a similar trend for **2 a** in toluene/MeCN mixtures (Figure 7b), where the peak occurs at somewhat higher polarity, near *f*(ϵ)=0.39. Our time‐resolved spectroscopy study only included solvents of high polarity, but based on this result (and the non‐exponential fluorescence decays in some solvents) we conclude that also in the extended rotor **2** two nonradiative decay pathways exist.

**Scheme 4:**
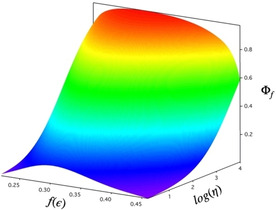
Dependence of the fluorescence quantum yield of **1** on solvent polarity and viscosity (schematic).

The solvent‐polarity dependence of the nonradiative decay rate affects the application of probe molecules such as **1** and **2** in several ways (Scheme 4). Near the polarity where the maximum fluorescence quantum yield is found, the nonradiative decay rate due to the twisting motions is relatively small, and the dynamic range of the application of the viscosity probe is smaller than in solvents in which the twisting rate is high. The higher barrier to rotation may, however, reduce the sensitivity to solvent viscosity, as was recently found for BODIPY molecular rotors.^[36]^ Finally, when two measurements are compared in which viscosity and polarity both change it may be difficult to disentangle the two effects on the fluorescence quantum yield or lifetime.

### 2.4 Effect of Viscosity on the Fluorescence Intensity

Fluorescence quantum yields of chromophores **1 a** and **2 a** exhibit pronounced sensitivity towards the viscosity of their micro‐environment. We measured the relative fluorescence quantum yields as a function of temperature from 283 to 328 K in glycerol. Viscosity values *η* were calculated as in ref. [37] According to the Förster‐Hoffmann relation^[38]^ the plots of log(I/I_0_) vs log(*η*/*η*_0_) should give straight lines with slopes α≈0.67. We find α=0.58 for **1 a** and α=0.53 for **2 a** (Figure S4 in SI). In our previous work we found α=0.67 for **1 a** when the viscosity of acetonitrile solutions was varied using pressure. The small difference with the value found here may be due to the neglect of the intrinsic barrier for the nonradiative decay process when the viscosity of glycerol is varied using temperature.

### 2.5 Surface Immobilization and Detection of Mechanical Contact

**Figure 8:**
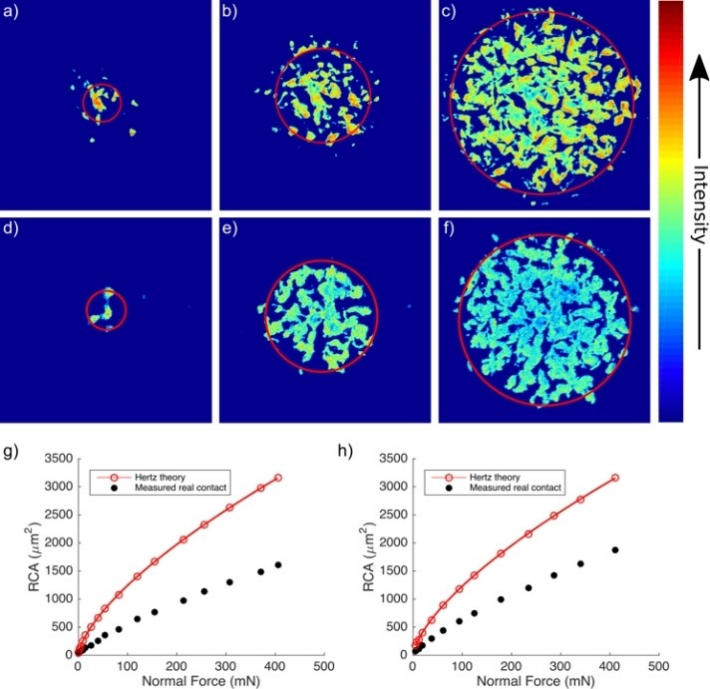
a)–c) Fluorescence intensity images of contact between **M1** (monolayer of immobilized **1 b**) and a roughened polystyrene sphere, with normal forces of 1.5, 81.5 and 405.5 mN, respectively. d)–f) images of the contact of a similar polystyrene bead with **M2**, with normal forces of 4.0, 62.0 and 340.0 mN, respectively. Red circles are the contact areas for an ideal sphere, calculated with Hertz’ theory.^[39]^ Image size 70×70 μm. g), h) Real contact area and the predicted Hertz contact area as a function of normal force.^[16]^

Compounds **1 b** and **2 b** were immobilized on glass cover slips in monolayers denoted as **M1** and **M2**, respectively. We created mechanical contacts of polymer beads on the functionalized cover slips as reported previously, shown in Figure 8.[^15^, ^16^] The real contact area was determined via thresholding^[16]^ and its dependence on the normal force is found to be essentially the same for **M1** and **M2**, showing that the performance of the two different probes for the measurement of mechanical contacts is equivalent.

**Figure 9:**
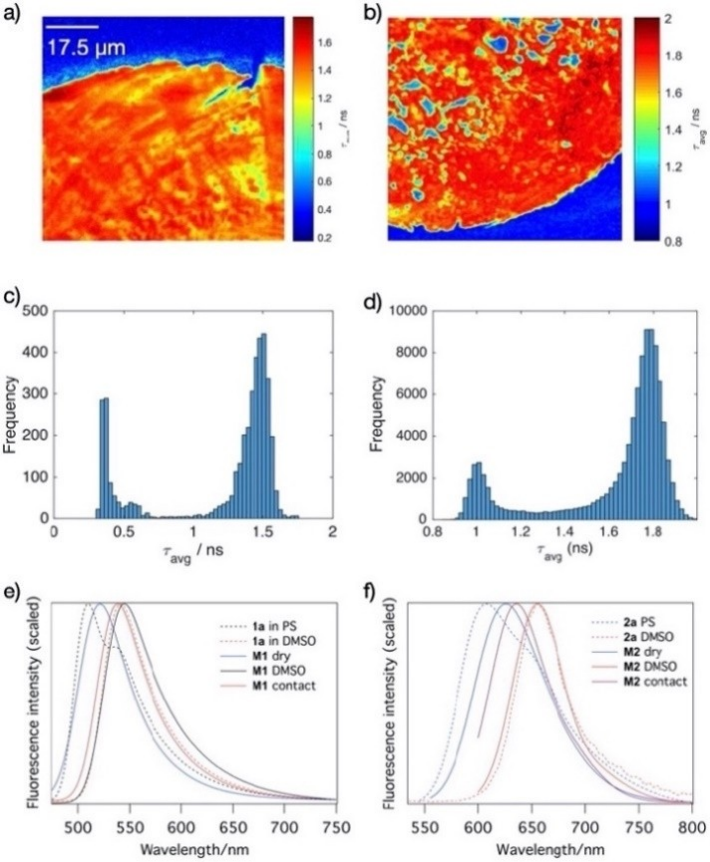
(a, b Fluorescence lifetime image of the contact area produced by pressing a smooth polystyrene bead onto DMSO‐wetted glass surfaces **M1** and **M2**. Color represents the average arrival time of the photons after the laser pulse at a measured point; (c, d) Histograms of lifetimes from images a and b; (e, f) Representative fluorescence emission spectra of **M1** and **1 a** and of **2 a** and **M2** in different environments.

To obtain more insight into the effect of the mechanical confinement on fluorescence, we made contact images using a fluorescence lifetime (FLIM) microscope (Figure 9 a,b). Two distinct populations (Figure 9c, d) can be observed near the contact zone, due to confined (within contact) and non‐confined (out of contact) molecular probes. Pixels that lie outside the contact zone (dark blue regions in Figure 9a and b) are characterized by average lifetime values around 0.4 ns for **M1**, and around 1 ns for **M2**. Within the contact zone, average lifetime values are narrowly distributed around 1.5 ns for **M1** and 1.8 ns for **M2**. The lifetime histograms show a clear separation in two populations, which is the basis for the thresholding that is used to determine the real contact area.

Figure 9e shows examples of fluorescence emission spectra of **1 a** measured in different environments. As the polarity of the local environment increases, fluorescence emission spectra shift towards longer wavelengths due to the solvent induced stabilization of the excited state. In low polarity polystyrene matrix the emission maximum is located at 510 nm. The immobilized version of **1** on glass (**M1**) has its emission maximum at 522 nm. Addition of DMSO on **M1** results in a pronounced red shift of emission to 541 nm. The emission maximum of **1 a** in DMSO is at 542 nm, which indicates that the immobilized probe is fully solvated in the case of DMSO wetted **M1**. Spectra obtained from the contact zone upon pressing a PS sphere onto a DMSO wetted **M1** show only a slight hypsochromic shift (∼5 nm) relative to the spectrum of **M1** in DMSO.

Immobilized **2 b** samples (**M2**) show behavior similar to that of **M1**, but we found that fluorescence lifetimes depend somewhat on details of sample preparation (acylation and surface functionalization time). We tentatively attribute this to self‐quenching at high grafting densities,^[40]^ because the fluorescence quantum yield of this chromophore is known to be concentration dependent in polymer matrices.^[20]^ Here we report quantities for samples prepared according to the procedure described in the experimental section. A typical fluorescence lifetime image, image histogram, and representative fluorescence emission spectra are shown in Figure 9b, d and f, respectively. Fluorescence lifetimes of **M2** in contact (τ_avg_∼1.8 ns) are somewhat longer than those of **M1**, but they still do not approach the lifetimes measured for the completely confined probe (∼2.4 ns). Fluorescence emission spectra shift similarly to those of **M1**, but spectral shifts seem to be more pronounced due to the higher degree of conjugation which results in a larger excited‐state dipole moment, in agreement with the larger solvatochromic effect for chromophore **2** than for **1**.

## 3 Conclusions

The experimental data presented here confirm the proposed model for the excited state dynamics of molecular rotor **1** in a range of solvents (Scheme 2), in particular by the unambiguous observation of the TICT intermediate in polar solvents. Such an intermediate is not observed in the transient absorption data of the π‐extended rotor **2**, probably because it decays faster than it is formed. Nonetheless, the solvent polarity dependent excited state decay times, and the solvent polarity dependent fluorescence intensities show that **2** behaves in essentially the same way as **1**. Immobilized rotor **2** can be excited and detected at longer wavelengths, which can be a practical advantage over using **1**. On the other hand, it has a somewhat smaller dynamic range, because the non‐radiative decay at low viscosity is not as fast as in **1**, both in solution and when immobilized. In imaging of mechanical contacts, the performances of **1** and **2** are not much different.

The barriers to rotation along the two available twisting coordinates show opposite solvent polarity dependence. As a result, a relatively intense fluorescence is observed in a range of solvents of low to medium polarity because both barriers are relatively high under those conditions. When applying this type of molecular probe, this effect should not be overlooked.

Fluorescence lifetime imaging and spectroscopy of the probes in wetted contacts show that the liquid used, in the present case DMSO, is still capable of rapid reorientation, stabilizing the polar excited state, despite the reduced mobility on the nanometer length scale, which causes the nonradiative decay via the TICT state to be slowed down. The fluorescence decay time of immobilized **1** and **2** in contact is much longer than in fluid solution, but shorter than in a glassy polymer matrix. The local environment of the probes in the confined state in contact is best described as a viscous liquid.

## Experimental Section

All experimental details are given in the Supporting Information.

## Conflict of interest

The authors declare no conflict of interest.

## Supporting information

As a service to our authors and readers, this journal provides supporting information supplied by the authors. Such materials are peer reviewed and may be re‐organized for online delivery, but are not copy‐edited or typeset. Technical support issues arising from supporting information (other than missing files) should be addressed to the authors.

Supplementary
