# Supplementary material for: Photophysics of Fluorescent Contact Sensors Based on the Dicyanodihydrofuran Motif
Source: Chemphyschem. 2020 Dec 4;22(2):221–7. doi: 10.1002/cphc.202000860 (PMC7898878; doi:10.1002/cphc.202000860)
Supplement: Supplementary file 1 — Supplementary [file CPHC-22-221-s001.pdf]

ChemPhysChem

Supporting Information

Photophysics of Fluorescent Contact Sensors Based on the Dicyanodihydrofuran Motif

Tomislav Suhina, Daniel Bonn, Bart Weber, and Albert M. Brouwer*

Synthesis and Characterization

Preparation of compounds **1a** [1] and **1b**[2] has been described previously. Compounds **2a** and **2b** were prepared by a procedure analogous to the one reported by Han et al.[3], using the appropriate dialkylaminobenzaldehyde. All reactions were conducted under N_2. Compound **2a**: 4-dimethylaminobenzaldehyde (675 mg, 4.53 mmol) and 2-(3-cyano-4,5,5-trimethylfuran-2(5H)-ylidene)malononitrile (388 mg, 2.06 mmol) were dissolved in 18 mL of pyridine, and 3 drops of acetic acid were added. The mixture turned dark blue after approximately 10 minutes of stirring at room temperature. After 48 hours, the mixture was poured into 150 mL of ice-water and filtered. The precipitate was dissolved in CH_2Cl_2, washed thoroughly with water/brine and the organic solvent was evaporated. The obtained dark blue powder was reprecipitated from CH_2Cl_2/MeOH to give 211 mg of **2a** (31 %). A similar preparation of this compound can be found in reference [4]. ^{1}H NMR (400 MHz, $CDCl_3$): (ppm) 1.76 (6H, s), 3.15 (6H, s), 6.70-6.78(3H, m), 7.53-7.63 (3H, m). ^{13}C NMR (400 MHz, $CDCl_3$): (ppm) 26.79, 40.21, 53.42, 96.75, 108.84, 111.42, 111.85, 112.17, 112.63, 121.85, 132.18, 148.34, 153.75, 174.20, 176.23. CSI-MS: MH+ 331.38 (calculated), 331.15 (observed).

Compound **2b**: A mixture of 4-(bis(2-hydroxyethyl)amino)benzaldehyde (778 mg, 3.72 mmol) and 2-(3-cyano-4,5,5-trimethylfuran-2(5H)-ylidene)malononitrile (340 mg, 1.69 mmol) in pyridine (200 mL) was stirred at room temperature until all reagents dissolved. After that, 3 drops of acetic acid were added to this mixture, which turned dark after 10 minutes of stirring at room temperature. The reaction mixture was left to stir for 24 hours, after which it was poured into 150 mL of ice-water. 0.1 M HCl was added until a precipitate could be observed. This precipitate was filtered, and 140 mg of blue powder with a reddish shine was obtained (22 %). Preparation of this compound is also described in ref. [5]. ^{1}H NMR (400 MHz, DMSO-d_6): (ppm) 1.76 (6H, s), 3.61 (8H, s), 6.7-6.9 (3H, m), 7.6-8 (3H, m). CSI-MS: MH+ 391.44 (calculated), 391.18 (observed).

Glass silanization

Cleaning: A Teflon rack with 4 borosilicate glass cover slips (2 cm × 2 cm × 170 µm) was sonicated in aqueous Extran solution (0.3 % w/w) for 30 mins, then sonicated in deionized water (10 min) and in ethanol (30 mins), dried under an air current, and placed in an ozone photoreactor for 2 hours.

Silanization with 3-aminopropyltrimethoxysilane (APTES): 80 mL of aqueous ethanol (96 %, v/v) was mixed with a small amount of acetic acid (so that pH = 5). To this mixture, APTES (2 mL) was added. Cover slips were placed in this solution and were left to react (with stirring) for 25-30 minutes. After this time, the cover slips were sonicated in ethanol 2 times for 30 minutes, dried in air, and annealed at 130 °C for 3 days.

Immobilization of 1b and 2b

Monolayer **M1**: **1b** (15 mg, 0.037 mmol) was dissolved in 80 mg of dry dimethylformamide (DMF) under N_2. To this solution, (benzotriazol-1-yloxy)tris(dimethylamino)phosphonium hexafluorophosphate (BOP; 100 mg, 0.230 mmol) and hydroxybenzotriazole (HOBt; 35 mg, 0.23 mmol) were added. 120 µL of N,N-diisopropylethylamine (DIPEA) were added to this solution, and a rack with cover slips (previously functionalized with APTES) was immersed in the solution. This was left to stir overnight. After that, cover slips were sonicated in ethanol twice for 15 minutes, and dried under an air current. Cover slips were stored in plastic containers until use.

Monolayer **M2**: Compound **2b** (10 mg, 0.025 mmol) was dissolved in dry dichloromethane under N_2 atmosphere. To this solution, 15 % phosgene solution (0.50 mL, 0.66 mmol) in toluene was added. This solution was stirred for 5 minutes in order to provide enough time for the acylation reaction of phosgene with the alcohol groups to take place, after which a rack with APTES silanized cover slips was added. This was stirred for approximately 20 minutes after which the cover slips were taken out and thoroughly washed in dichloromethane, toluene and ethanol by sonication for 15 minutes in each solvent. Samples were stored in sealed plastic containers under Ar until use.

Surface density of molecular rotors

In order to estimate the grafting density of chromophores on our functionalized surfaces, we measured UV-Vis absorption spectra (Figure S1). Absorbance is very low, as expected from a single dye layer attached to the surface. Compared to spectra of **1** and **2** in solutions, absorption spectra of **M1** and **M2** appear to be broader. If we nonetheless assume that the optical cross sections of immobilized molecules are the same as in toluene solutions (ε = 62200 $M^{-1}cm^{-1}$ and ε = 42300 $M^{-1}cm^{-1}$ for **1a** and **2a**, respectively), Lambert-Beer law can be used to estimate the number of molecules per unit area to be ~$A/(2\varepsilon)$ (since both sides of cover slips are functionalized). The grafting density obtained in this way is ~1 molecule per 10 nm^2 for both **M1** and **M2**. Such a low grafting density should, in principle, not allow for a large degree of homo FRET and self quenching.

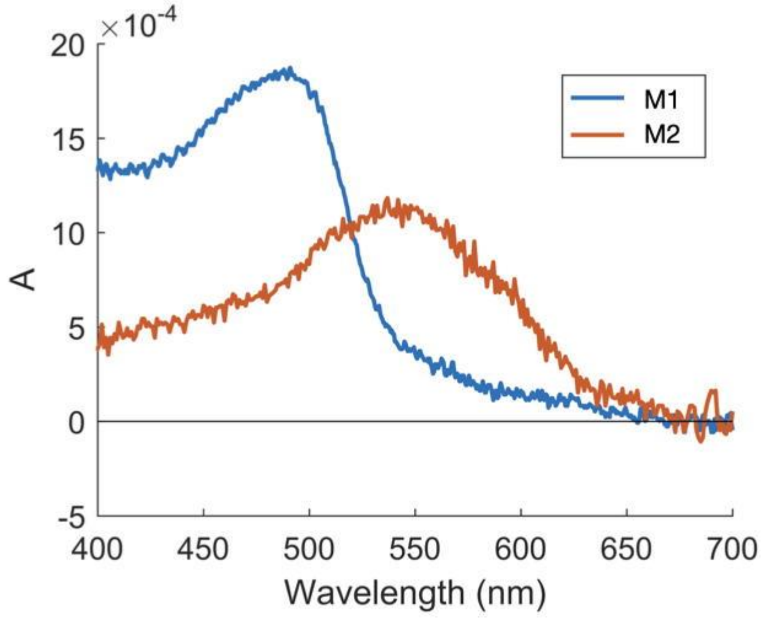

Figure S1. Electronic absorption spectra of cover slips used in contact imaging.

Polystyrene beads

Polystyrene spheres (microbeads) were purchased from the Precision Plastic Ball Company (Addingham, Ilkley, UK) and roughened by placing them over 240 grit sand paper on an orbital shaker (1200 rpm) for 2 days.

Photophysical measurements.

Almost all equipment and methods used for spectroscopic experiments and microscopy has been described in earlier work.[2,6–9] For analysis of the transient absorption data we used Glotaran 1.5.1.[10]

Fluorescence lifetime images were measured with a Olympus IX-71 microscope equipped with a MicroTime 200 TCSPC unit (PicoQuant GmbH) and a 100x 1.4 NA objective (UplanSApo, Olympus), mounted on a piezo-scanning stage (Physik Instruments GmbH). A detection pinhole with a diameter of 75 μm was used. An NKT Photonics SuperK Extreme Supercontinuum white laser (80 MHz) was used as the excitation source. Because of the nonexponential nature of the fluorescence decays, the average fluorescence lifetimes are defined as the average times that emitted photons take to reach the detector after the excitation pulse.

Fluorescence spectra of **M1** and **M2** (figure 9 in main text) were obtained by passing the emitted light in the confocal microscope to a spectograph (Spectra Pro-150, *Acton Research Corp.*) equipped with an EM-CCD camera (PhotonMax 512B, *Roper Scientific*).

Additional Figures and Tables

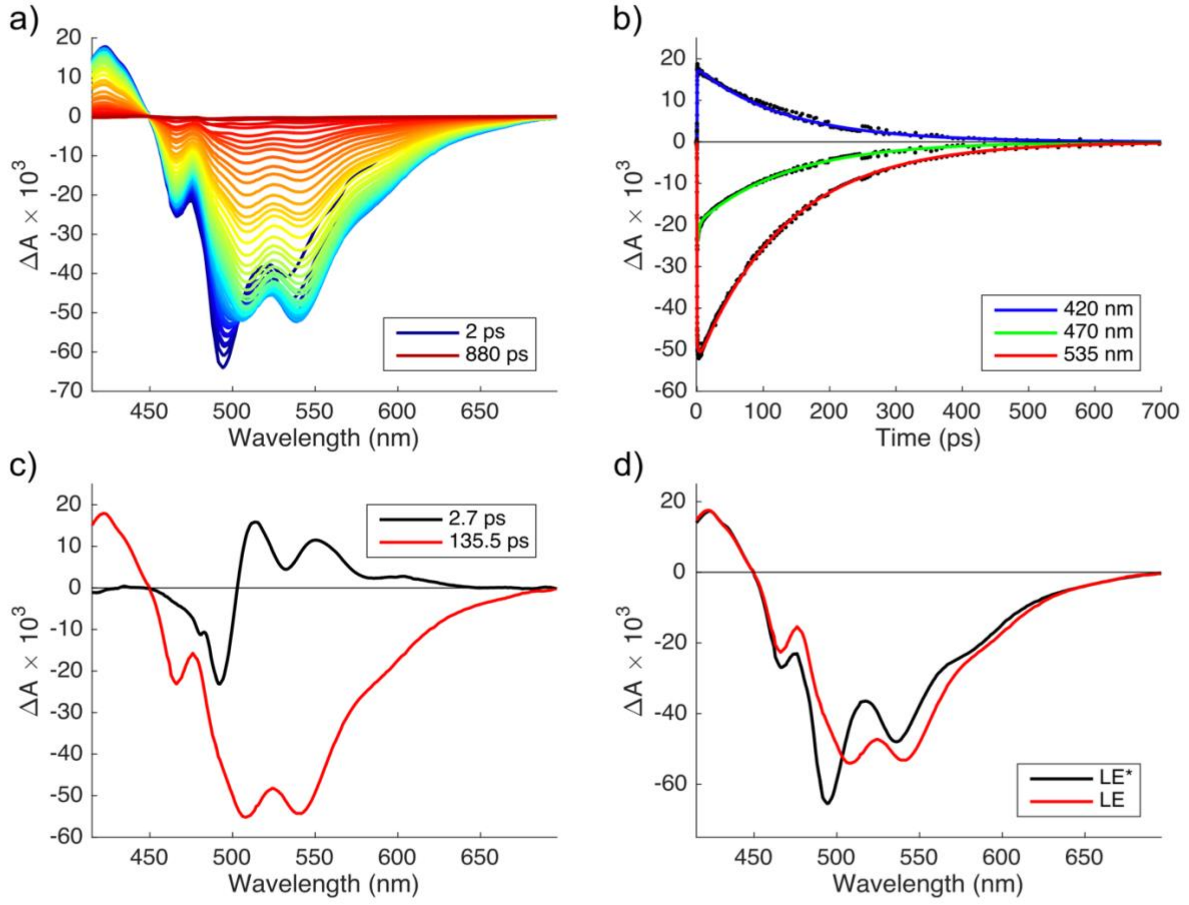

Figure S2. Transient absorption spectra of **1a** in toluene. a) transient spectra at different delay times; b) selected time traces (black markers) and fits (colored lines) produced by compartmental global analysis; c) decay-associated difference spectra; d) species-associated spectra.

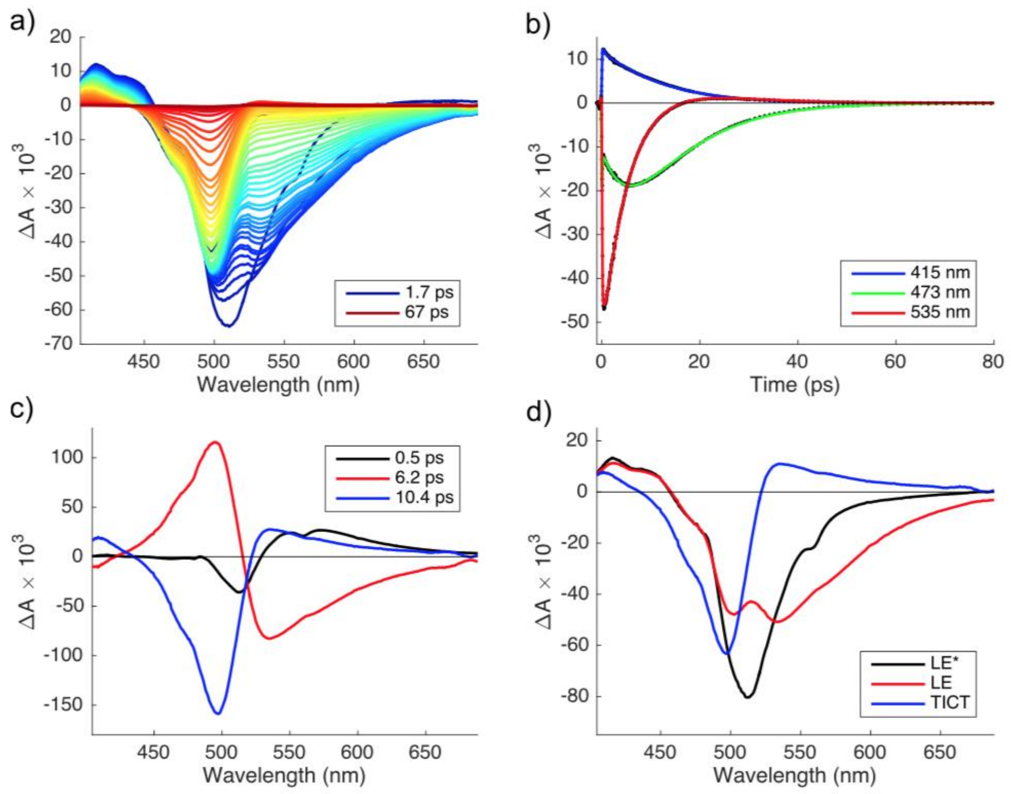

Figure S3. Vis-pump / vis-probe probe measurements for **1a** in MeCN: a) selected transient spectra; b) selected time traces (black markers) and fits (colored lines) produced by compartmental global analysis; c) decay-associated difference spectra (with time constants); d) species-associated difference spectra.

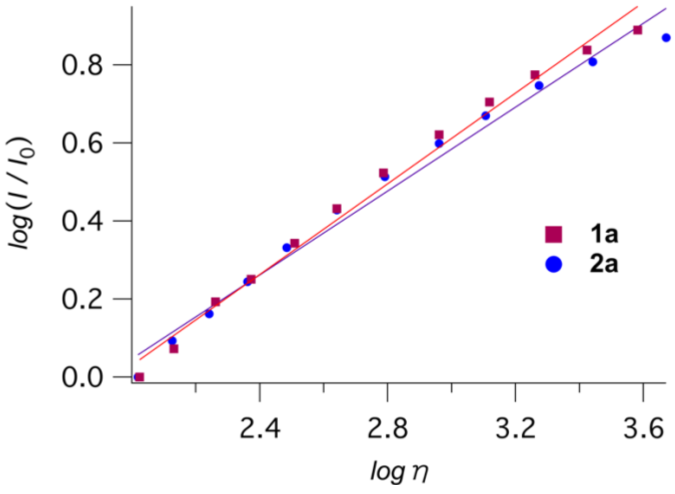

Figure S4. Förster-Hoffmann plot for **1a** and **2a** in glycerol. The viscosity was varied by varying the temperature.[11]

Table S1. Data from time-resolved fluorescence measurements (TCSPC) of **2a**.

solvent	τ_1 (ps)	A_1	τ_2 (ps)	A_2	χ^2
toluene	107	1.00			1.01
dioxane	217	1.00			1.15
propanol	35	0.24	154	0.76	1.15
butanol	40	0.25	226	0.75	1.11
cyclohexanol	237	0.37	833	0.63	1.13
methanol	28	1.00			1.73
MeCN	26	1.00			2.31
DMF	57	1.00			1.33
DMSO	3[a]	0.56	70	0.44	1.27

[a] fixed at value from transient absorption experiment

References

[1] U. Gubler, M. He, D. Wright, Y. Roh, R. Twieg, W. E. Moerner, *Adv. Mater.* **2002**, *14*, 313-317.

[2] T. Suhina, B. Weber, C. E. Carpentier, K. Lorincz, P. Schall, D. Bonn, A. M. Brouwer, *Angew. Chem. Int. Ed.* **2015**, *54*, 3688-3691.

[3] L. Han, Y. Jiang, W. Li, Y. Li, J. Liao, *Mater. Lett.* **2008**, *62*, 1495-1498.

[4] X. Tang, L. Pan, K. Jia, X. Tang, *Chem. Phys. Lett.* **2016**, *648*, 114-118.

[5] J. Pérez-Moreno, Y. Zhao, K. Clays, M. G. Kuzyk, Y. Shen, L. Qiu, J. Hao, K. Guo, *J. Am. Chem. Soc.* **2009**, *131*, 5084-5093.

[6] T. Suhina, S. Amirjalayer, B. Mennucci, S. Woutersen, M. Hilbers, D. Bonn, A. M. Brouwer, *J. Phys. Chem. Lett.* **2016**, *7*, 4285-4290.

[7] T. Suhina, S. Amirjalayer, S. Woutersen, D. Bonn, A. M. Brouwer, *Phys. Chem. Chem. Phys.* **2017**, *19*, 19998-20007.

[8] B. Weber, T. Suhina, T. Junge, L. Pastewka, A. M. Brouwer, D. Bonn, *Nat. Commun.* **2018**, *9*, 888-1-7.

[9] B. Weber, T. Suhina, A. M. Brouwer, D. Bonn, *Sci. Adv.* **2019**, *5*, eaav7603.

[10] J. J. Snellenburg, S. P. Laptenok, R. Seger, K. M. Mullen, I. H. M. van Stokkum, *J. Stat. Softw.* **2012**, *49*, 1-22.

[11] N.-S. Cheng, *Ind. Eng. Chem. Res.* **2008**, *47*, 3285-3288.
